# Impact of health literacy, social support, and socioeconomic position on the serum uric acid level in asymptomatic hyperuricaemia patients in China: a structural equation model

**DOI:** 10.1186/s12889-024-19085-6

**Published:** 2024-06-17

**Authors:** Yunfang Jing, Lilai Ma, Yuanfan Zhang, Xiaohong Li, Jun Jiang, Jie Long, Ling Ma

**Affiliations:** 1 Suining First People’s Hospital, Sichuan Province, Suining, 629000 China; 2 Department of Nutrition and Food Hygiene, School of Public Health, Southwest Medical University, Sichuan Province, Luzhou, 646000 China; 3 Health Management Center, The Affiliated Hospital, Southwest Medical UniversitySichuan Province, Luzhou, 646000 China

**Keywords:** Hyperuricaemia, Healthy literacy, Serum uric acid, Social support, Socioeconomic position

## Abstract

**Background:**

Hyperuricaemia (HUA) poses a significant public health challenge on a global scale. It is mostly asymptomatic hyperuricemia (AHU) with unsatisfactory recognition and control rates. The role of health literacy in influencing health outcomes is of utmost importance, and enhancing health literacy is helpful for patients in managing risk factors. Additionally, social support and socioeconomic position (SEP) have been identified as potential factors influencing health. However, the exact relationships between these factors and AHU remain unclear. This study aimed to investigate the status of health literacy among patients with AHU and explore the relationships between health literacy, social support, SEP, and serum uric acid (SUA) levels.

**Methods:**

A cross-sectional study was conducted among 349 participants with AHU in Luzhou, China. The research instruments included a sociodemographic characteristics questionnaire, the Health Literacy Scale for Chronic Patients (HLSCP), and the Social Support Scale (SSRS). The construction of the SEP index was achieved through the application of principal component analysis. Univariate and hierarchical regression analyses were used to evaluate the associations between SEP, social support, health literacy, and SUA levels. Furthermore, structural equation modelling (SEM) was utilized to examine these associations.

**Results:**

(1) Most patients exhibited low health literacy (90.18 ± 15.11), and only 44.4% possessed basic health literacy. (2) SEP was positively correlated with SUA levels (*β* = 4.086, *P* < 0.001), and health literacy was negatively related to SUA levels (*β* = -0.399,* P* < 0.001). There was no significant relationship between social support and SUA levels (*β* = 0.051, t = 1.085). (3) Health literacy mediated the association between SEP and SUA levels (*β* = -0.490, 95% CI: -0.620 to -0.382). SEP had a direct positive effect on SUA levels (*β* = 0.723) and health literacy (*β* = 0.696), and the total effect of SEP on SUA levels was 0.233.

**Conclusions:**

The findings indicate a low level of health literacy among patients with AHU and suggest that health literacy might play a mediating role in the relationship between SEP and SUA levels. Consequently, future initiatives are recommended to prioritize health literacy and devise appropriate intervention strategies to enhance the self-management capabilities of patients with AHU.

**Supplementary Information:**

The online version contains supplementary material available at 10.1186/s12889-024-19085-6.

## Introduction

HUA is a chronic metabolic disorder resulting from an imbalance in purine metabolism characterized by SUA levels exceeding 420 μmol/L (7.0 mg/dL) regardless of gender [1]. AHU is characterized by elevated SUA levels and no gout-related clinical symptoms (including gouty arthritis, uric acid nephropathy, urinary calculus, etc.) The prevalence of AHU is 17.4% in mainland China [2], 17.6% in the United States [3], and 16.3% in Spain [4]. AHU has become a global public health concern, with prevalence increasing with age, higher in men than in women, and the incidence tends to be younger [5]. Due to the absence of apparent symptoms, patients with AHU often lack awareness of the proper management of their condition, resulting in low rates of attention (35.29%) and control (8.0%) [6, 7]. Previous studies have shown that urate is related to gout and uric acid nephropathy [8]. Recent studies have also demonstrated an association between elevated SUA levels and the risk of coronary heart disease [9], diabetes [10], hypertension [11], and chronic kidney disease [12]. Therefore, it is crucial to control SUA levels in AHU patients to alleviate the burden of this disease.

The SUA levels of AHU patients are related not only to disease-related factors but also to other factors, such as health literacy, social support, and SEP.

Health literacy is defined as the “ability of a person to obtain, process, and comprehend the fundamental health information and services needed to make health decisions” [13]. Adequate health literacy could help individuals obtain the knowledge, abilities, and confidence to improve their lifestyle and manage disease. Inadequate health literacy might contribute to adverse health outcomes, such as being more likely to have a higher comorbidity prevalence, and being at risk for chronic kidney disease onset [14–16]. Previous studies have shown that chronic conditions are associated with health literacy, and improving health literacy may be a prospective method to help patients better manage their health [17]. The literature shows that the main reason for the discontinuation of urate-lowering drugs among gout patients is poor health literacy [18] and suggests that health professionals utilize online booklets to increase the health literacy of these patients for controlling SUA levels [19]. However, health literacy has not been examined in AHU patients.

Social support is defined as the “social resources that persons perceive to be available or that are provided to them” [20]. Studies have demonstrated that greater social support is associated with better self-rated health and fewer health-impairing, such as having lower SUA levels [21, 22]. [19]Previous studies have shown that social support has a positive relationship with health literacy and has chain-mediating effects on education level, medication adherence [23], and self-management [24]. For gout patients, studies have shown that self-management behaviour is related to social support [25]. Evidence suggests that social support is an independent protective factor against HUA (OR = 0.944) [26]. Unfortunately, the relationship between health literacy, social support, and SUA levels in AHU patients in China has not been explored.

Moreover, SEP is another important factor influencing the SUA levels, as well as health literacy. Several studies have shown that a higher educational level is related to a higher risk of HUA, but a lower risk of gout [27]. Other studies corroborate that SEP factors can influence SUA levels and/or facilitate monosodium urate crystal formation [28]. A randomized controlled trial demonstrated that providing education improvement programs to AHU patients could significantly reduce SUA levels [29]. In the literature, a low SEP has been identified as a risk factor for low health literacy levels [30]. Education degree, monthly family average income, and occupation were positively associated with health literacy among adults [31].

**Fig. 1:**
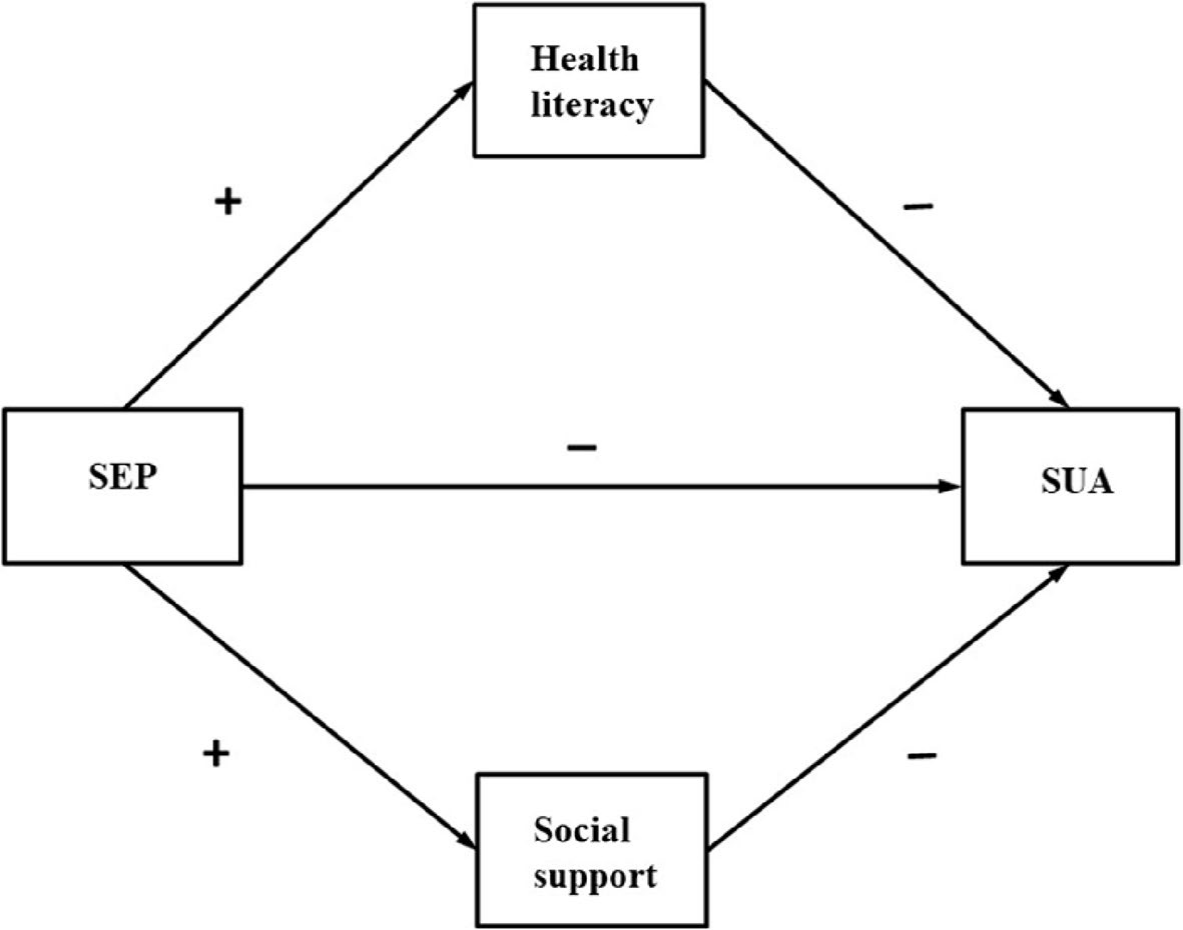
The theoretical model and hypotheses

According to the abovementioned literature, health literacy, social support, SEP, and health outcomes have complex relationships. However, how these variables and their combined effects affect AHU is still unknown, and no study has explored the mediating effect of health literacy on AHU patients using SEM. Therefore, this study aimed to investigate the health literacy status of AHU patients; explore the influence of health literacy, social support, and SEP on SUA levels; and validate the associations between health literacy, SEP indicators (education level, occupation type, and income), and SUA levels. Based on the existing literature, we hypothesized that (H1) SEP is related to SUA levels; (H2) health literacy has a mediating effect between SEP and SUA levels; and (H3) social support has a mediating effect between SEP and SUA levels. And we proposed a theoretical model (Fig. 1).

## Materials and methods

All procedures were performed in accordance with the Declaration of Helsinki, and the study was approved by the Ethics Committee of the Affiliated Hospital of Southwest Medical University (KY2022-175). All the research participants provided informed consent and were kept anonymous.

### Study design and population

We conducted a cross-sectional study from March 2022 to September 2022 among AHU patients in Luzhou City, Sichuan Province, China. The survey used a convenience sampling method to recruit participants from the Affiliated Hospital of Southwest Medical University Health Management Center and two community health service centres.

The inclusion criteria were as follows: (1) had a diagnosis of HUA according to the 2019 Chinese guidelines for the diagnosis and treatment of gout and HUA; (2) were > 18 years of age; (3) had the capacity (or with the help of the investigator) to understand the study and complete the required research questionnaire; (4) were conscious; and (5) voluntarily participated in this study and signed the informed consent.

The exclusion criteria were as follows: (1) had a diagnosis of gout; (2) had clinical symptoms related to gout, such as gouty arthritis or tophus; (3) had a psychotic disorder or disturbance of consciousness; (4) were pregnant; (5) had severe liver, kidney or other important organ dysfunctions, such as hepatic sclerosis and renal failure; and (6) had subsequent HUA (e.g., an inborn error of metabolism, myeloproliferative disorders, malignant tumour). The sample size was calculated according to the cross-sectional study formula $n\;=\frac{Z_{1\;-\alpha/2}^2P\;(1-P)}{d^2}$ [32], with a 95% confidence interval, 80% power, and health literacy possession rate. According to the preinvestigation results, 21.9% of the AHU patients had health literacy and an expected nonresponse rate of 20%. Finally, a sample size > 316 was obtained.

### Study measures

The questionnaire included questions about sociodemographic characteristics, disease-related information, and two scales selected according to the purpose of the research. Sociodemographic characteristics included age, gender, place of residence, residence type, marital status, and the ability to pay for medical payments. Disease-related information included the number of comorbidities and duration of AHU. An additional file shows this in more detail [see Additional file 1].

#### Socioeconomic position (SEP)

SEP was measured by education level, occupation type, and monthly family average income. Education level was categorized as follows: primary school or below, junior high school, senior high school or special secondary school, junior college, or a bachelor’s degree or higher [33]. According to the Family Socioeconomic Status Rating Scale compiled by Baoguo S and Jiliang S [34], occupation type was classified into 5 levels: casual workers, unemployed and agricultural labourers; manual workers and self-employed individuals; clerical workers; professional and technical staff; and senior managers. Patients reported their monthly family average income using 5 answer options (1 = less than or equal to 2000 RMB; 2 = 2001–3000 RMB; 3 = 3001–4000 RMB; 4 = 4001–5000 RMB; 5 = more than or equal to 5001 RMB). Principal component analysis was used to construct the SEP index. Bartlett’s test of sphericity was significant (*P* < 0.001), and the Kaiser–Meyer–Olkin (KMO) value was 0.688. The first component explained 77.383% of the total variation.

#### Health Literacy Scale for Chronic Patients (HLSCP)

In this study, a health literacy scale for chronic patients was used. The HLSCP was developed by Jordan et al. [35]. and translated by Sun [36]. It contains 24 items, and each item has five options ranging from 1 (with great difficulty or very unwilling) to 5 (with no problem or very willing). The ability to procure information, communication and interaction abilities, health improvement willingness, and economic support willingness are measured. The final score ranges from 24 to 120 points, with a higher score indicating a higher health literacy level. A score of 80% or more of the total score (≥ 96 points) is considered to possess basic health literacy [37]. In this study, the Cronbach’s alpha of the scale was 0.930, and the KMO value was 0.915, which indicated relatively good reliability and validity.

#### Social Support Rating Scale (SSRS)

 [38] The SSRS includes 10 items and has 3 dimensions: objective support, subjective support, and support utilization. The scale was developed and validated by the Chinese scholar Shuiyuan Xiao and is used to evaluate the types and levels of social support received by elderly people. The total score ranges from 12–66, and scores < 22, 23–44, and 45 and above represent low, medium, and high levels of social support, respectively. Higher scores indicate higher levels of social support. The Cronbach’s alpha of the overall scale in this study was 0.716.

### Data collection

Data collectors were professional masters of public health students who had been trained in field investigation courses. Before the investigation, all the investigators had received sufficient training to ensure they had good survey skills for data collection. Pre-surveys of 30 samples were conducted before the actual survey. During the formal survey, the purpose, significance, and content of the study were explained in detail, and one-on-one interviews were conducted with patients. If patients could not complete the questionnaire by themselves, they completed it with the help of investigators.

### Statistical analysis

The data were entered twice independently using EpiData software (version 3.0; EpiData Association, Odense, Denmark). The data analysis was performed using SPSS 26.0 (IBM SPSS Statistics, USA) and AMOS 24.0. The numerical variables are expressed as the mean (SD) for normally distributed data and as the median and interquartile range for nonnormally distributed data. The categorical variables are expressed as n (%) and were compared by the chi-square test. A principal component analysis (PCA) was used to construct the comprehensive SEP index. The associations among the study variables were analysed using the Spearman rank correlation method. Hierarchical regression models were used to analyse the effects of SEP, social support, and health literacy on SUA levels. The direct and indirect relationships among the variables were estimated by SEM and fit by the maximum likelihood estimation method. A *P* value less than 0.05 was considered to indicate statistical significance.

### Missing data

Variables with > 15% missing data were excluded from the analyses. A total of 381 questionnaires were sent out, and 349 valid questionnaires were collected; the validity rate was 91.60%. For categorical variables, missing data were replaced with the mode. For continuous variables, missing data were replaced with the mean and median (for nonnormally distributed data).

## Results

### Characteristics of patients with AHU

Overall, 349 patients with AHU were enrolled in this study; 292 (83.7%) were male, and 57 (16.3%) were female. The mean age was 49.15 $\pm$ 12.73 years, with the youngest participant being 20 years old and the oldest being 83 years old. The median SUA level was 526.00 μmol/L. Most respondents lived in urban areas (251, 71.9%), had a bachelor’s degree or higher (103, 29.5%), were professional and technical staff (153, 98.0%), and had a monthly family average income ≥ 5001 RMB (116, 33.2%). The characteristics of the participants are shown in Table 1.

**Table 1:** Demographic characteristics and their associations with social support and health literacy (*n* = 349)

Variable	Classification	n (%)	Have health literacy		χ^2^	*P*	Social support	*t/F*	*P*
			n	%					
Gender	Male	292 (83.7)	132	45.2	0.455	0.500	42.42 ± 6.88	1.807	0.072
	Female	57 (16.3)	23	40.4			40.60 ± 7.46		
Age (years)	< 45	124 (35.5)	73	58.9	17.428	< 0.001	43.01 ± 6.38	15.917	< 0.001
	45 - 60	166 (46.7)	64	38.6			43.06 ± 6.54		
	> 60	59 (16.9)	18	30.5			37.63 ± 7.81		
Residence	Rural	98 (28.1)	11	11.2	60.794	< 0.001	42.52 ± 5.64	0.747	0.456
	Urban	251 (71.9)	144	57.4			41.97 ± 7.46		
Residence type	Living alone	14 (4.0)	8	57.1	0.957	0.328	35.86 ± 8.23	-3.474	0.001
	Not living alone	335 (96.0)	147	43.9			42.39 ± 6.83		
Marital status	Married	303 (86.8)	134	44.2	0.033	0.856	42.98 ± 6.69	6.178	< 0.001
	Unmarried, divorced or widowed	46 (13.2)	21	45.7			36.48 ± 6.41		
Education level	Primary school or lower	61 (17.5)	3	4.9	104.986	< 0.001	38.3 ± 6.78	11.208	< 0.001
	Junior high school	81 (23.2)	21	25.9			40.17 ± 7.2		
	High school or Special Secondary	48 (13.8)	17	35.4			43.23 ± 6.95		
	Junior college	56 (16.0)	33	58.9			43.84 ± 5.62		
	Bachelor’s degree or higher	103 (29.5)	81	78.6			44.48 ± 6.36		
Occupation	Casual workers, unemployed individuals and agricultural labourers	150 (43.0)	26	17.3	92.588	< 0.001	39.72 ± 7.2	9.276	0.001
	Manual workers and self-employed individuals	35 (10.0)	16	45.7			41.43 ± 7.17		
	Clerical workers	4 (1.1)	1	25			46.00 ± 8.83		
	Professional and technical staff	153 (98.0)	106	69.3			44.44 ± 5.64		
	Senior managers	7 (2.0)	6	85.7			44.29 ± 10.83		
Monthly family average income	≤ 2000 RMB	50 (14.3)	4	8	81.132	< 0.001	35.56 ± 6.74	21.361	< 0.001
	2001–3000 RMB	42 (12.0)	16	38.1			40.67 ± 6.48		
	3001–4000 RMB	78 (22.3)	25	32.1			41.06 ± 6.60		
	4001–5000 RMB	63 (18.1)	22	34.9			44.06 ± 6.30		
	≥ 5001 RMB	116 (33.2)	88	75.9			44.87 ± 5.81		
The ability to pay for family medical costs	Unable to pay	24 (6.9)	3	12.5	89.323	< 0.001	38.46 ± 7.20	18.555	< 0.001
	Can barely pay	142 (40.7)	27	19.0			40.12 ± 6.98		
	Able to pay	183 (52.4)	125	68.3			44.16 ± 6.35		

The health literacy and social support status of the AHU patients are also shown in Table 1. The results of the chi-square test, independent t-tests, and ANOVA showed that young age (*P* < 0.001), higher education (*P* < 0.001), and higher monthly family average income (*P* < 0.001) were associated with a high proportion of health literacy and high social support scores. The proportion of patients with health literacy was significantly greater in the rural group than in the urban group (*P* < 0.001). Social support was significantly greater in the group that was not living alone or married than in the group that was living alone or was unmarried, divorced, or widowed.

The total scores for health literacy and social support and their domains in AHU patients are shown in Table 2. The mean health literacy score of the participants was 90.18 $\pm$ 15.11, and 155 (44.4%) participants had health literacy. The mean score for social support was 42.15 $\pm$ 7.00, 3 (0.9%) patients were in the poor social support group, 197 (56.4%) patients were in the suitable social support group, and 149 (42.7%) patients were in the excellent social support group.

**Table 2:** Total social support and health literacy scores and their dimensions (*n* = 349)

Variable	Classification	Mean $\pm$ SD/frequency (%)
Health literacy and its dimensions	Information acquisition ability (9–45)	38.74 $\pm$ 8.05
	Communication interaction ability (9–45)	27.10 $\pm$ 7.32
	Health improvement willingness (4–20)	16.05 $\pm$ 1.64
	Economic support willingness (2–10)	8.29 $\pm$ 1.97
	Total health literacy score (24–120)	90.18 $\pm$ 15.11
Health literacy level	Possesses health literacy	155 (44.4%)
	Does not possess health literacy	194 (55.6%)
Social support and its dimensions	Objective support (1–22)	9.53 $\pm$ 2.68
	Subjective support (8–32)	25.50 $\pm$ 5.13
	Support utilization (3–12)	7.09 $\pm$ 2.07
	Total social support score (12–66)	42.15 $\pm$ 7.00
Social support level	Poor (≤ 22)	3 (0.9%)
	Suitable (23–44)	197 (56.4%)
	Excellent (≥ 45)	149 (42.7%)

### Correlations between study variables

**Fig. 2:**
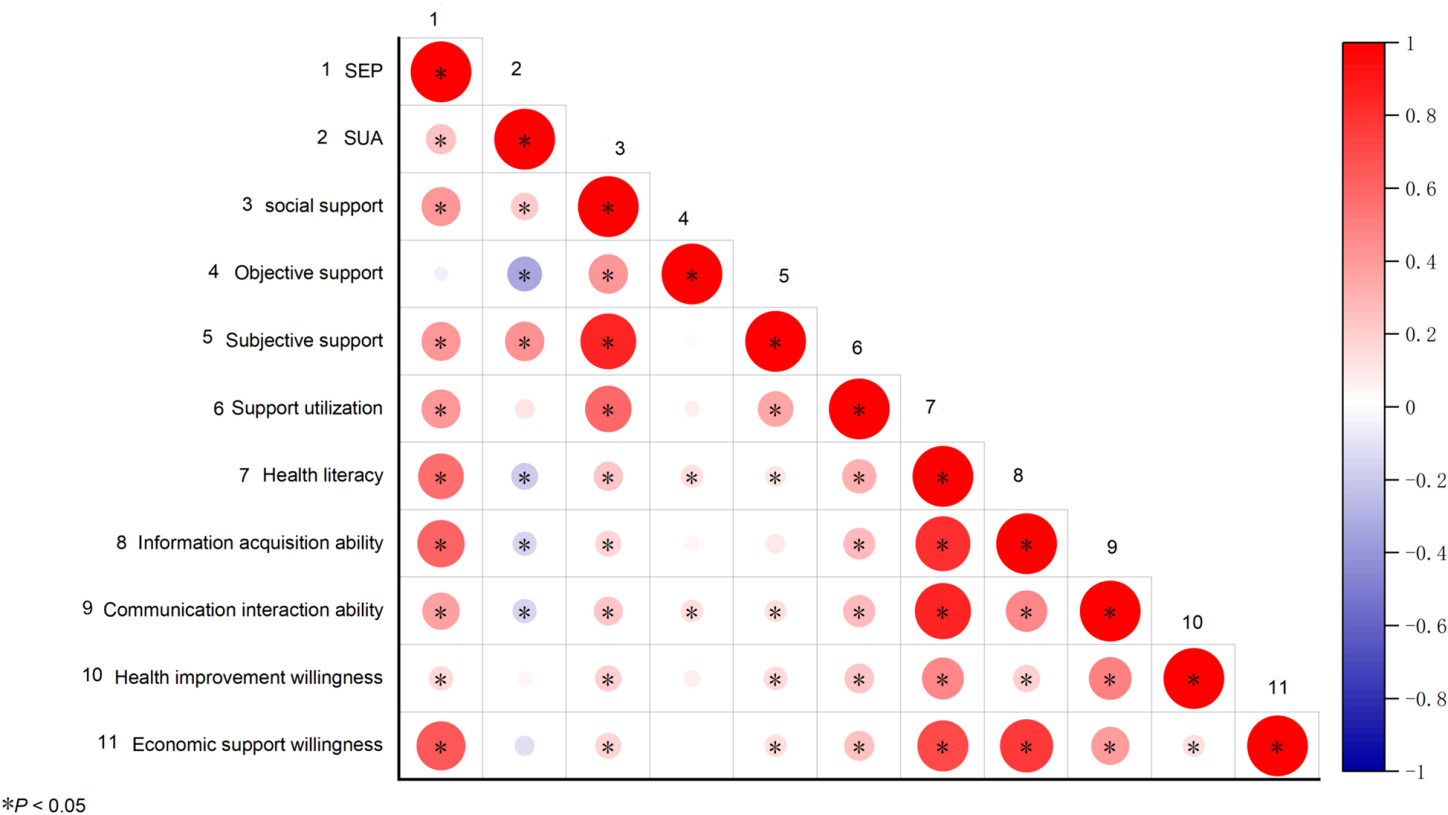
Correlation matrix for the study variables: the red and blue dots represent positive and negative correlations, respectively. Small dots with light colours signify correlations of lesser intensity, whereas larger dots with darker hues signify correlations of higher intensity

Spearman's correlations for SEP, SUA levels, social support, and health literacy are graphically displayed in Fig. 2. The correlation coefficients and significance levels are shown in Table 3. SEP (r = 0.245,* P* < 0.05) and social support (r = 0.205,* P* < 0.01) were positively associated with SUA levels, while health literacy was negatively correlated with SUA levels (r = -0.190, *P* < 0.01). SEP was positively correlated with social support (r = 0.412, *P* < 0.01) and health literacy (r = 0.566, *P* < 0.01), and social support was positively correlated with health literacy (r = 0.230, *P* < 0.01).

**Table 3:** Spearman’s correlation matrix of the study variables

Variable	1	2	3	4	5	6	7	8	9	10
SEP										
SUA levels	0.245^*^									
Social support	0.412^**^	0.205^**^								
Objective support	-0.048	-0.320^**^	0.414^**^							
Subjective support	0.412^**^	0.417^**^	0.845^**^	0.023						
Support utilization	0.409^**^	0.104	0.587^**^	0.065	0.345^**^					
Health literacy	0.566^**^	-0.190^**^	0.230^**^	0.142^**^	0.107^*^	0.317^**^				
Information acquisition ability	0.603^**^	-0.159^**^	0.178^**^	0.055	0.098	0.272^**^	0.814^**^			
Communication interaction ability	0.372^**^	-0.155^**^	0.224^**^	0.140^**^	0.123^*^	0.278^**^	0.851^**^	0.462^**^		
Health improvement willingness	0.157^**^	0.056	0.180^**^	0.082	0.146^**^	0.234^**^	0.479^**^	0.200^**^	0.499^**^	
Economic support willingness	0.644^**^	-0.104	0.176^**^	0.021	0.128^*^	0.245^**^	0.711^**^	0.774^**^	0.395^**^	0.136^*^

^*^P < 0.05, **P < 0.01

### Hierarchical regression analyses

Table 4 shows the results of the hierarchical regression model. This model was constructed to determine the associations of SEP, social support, and health literacy with SUA levels in AHU patients. In Model 1, age, gender, and AHU duration were added as control variables. In Model 2, SEP was added, allowing the effects of SEP to be considered in the second step after accounting for control variables. The results showed that the relationship between SEP and SUA levels was not statistically significant (*β* = 0.017, *P* > 0.05). In Model 3, social support and health literacy were incorporated into the third hierarchy to determine whether they explained any variance beyond that explained by SEP. The results showed that SEP was positively correlated with SUA levels (*β* = 4.086,* P* < 0.001) and that health literacy was negatively related to SUA levels (*β* = -0.399,* P* < 0.001); however, there was no significant relationship between social support and SUA levels (*β* = 0.051, t = 1.085). The variance explained by the total model was 38.2% (F = 35.203, *P* < 0.001).

**Table 4:** Results of hierarchical regression analysis

Variable	Model 1			Model 2			Model 3		
	*B*	*β*	*t*	*B*	*β*	*t*	*B*	*β*	*t*
Constant	588.854	-	23.723***	586.346	-	22.409***	825.494	-	15.346***
Age	-1.714	-0.194	-4.111***	-1.645	-0.186	-3.478**	-1.510	-0.171	-3.428**
Gender	-14.479	-0.048	-1.004	-14.663	-0.048	-1.015	-14.819	-0.049	-1.103
HUA duration	15.920	0.481	10.457***	42.409	0.377	7.604***	12.928	0.391	8.370***
SEP				0.946	0.017	0.307	14.644	0.256	4.086***
Social support							0.823	0.051	1.085
Health literacy							-2.973	-0.399	-7.454***
*R* ^2^	0.280			0.280			0.382		
∆*R*^2^	0.280			0.000			0.101		
*F*	44.754***			33.501***			35.203***		

^*^P < 0.05, ** P < 0.01, ***P < 0.001

### Construction and testing of the SEM

According to the research hypothesis, a preliminary model is established. The relationship between social support and SUA levels was not statistically significant. Therefore, we eliminate this path, as shown in Fig. 2. The maximum likelihood method is used to estimate the parameters and modify the model. The final model fit values showed that the relative chi-square value was 2.872, the goodness-of-fit index was 0.982, the adjusted goodness-of-fit index was 0.945, the comparative fit index was 0.984, the normed fit index was 0.976, the Tucker–Lewis index was 0.965, the incremental fit index was 0.984 and the root-mean-squared error of approximation was 0.073, suggesting a relatively satisfactory model fit to the data (Table 5).

**Table 5:** Model fit indices

Variable	χ^2^/df	GFI	AGFI	CFI	NFI	TLI	IFI	RMSEA
Fit index	2.872	0.982	0.945	0.984	0.976	0.965	0.984	0.073
Reference value	< 5.00	> 0.90	> 0.90	> 0.90	> 0.90	> 0.90	> 0.90	< 0.08

*GFI* Goodness-of-fit index, *AGFI* Adjusted GFI, *CFI* Comparative fit index, *NFI* Normed fit index, *TLI* Tucker–Lewis index, *IFI* Incremental fit index, *RMSEA* Root mean square error of approximation

**Fig. 3:**
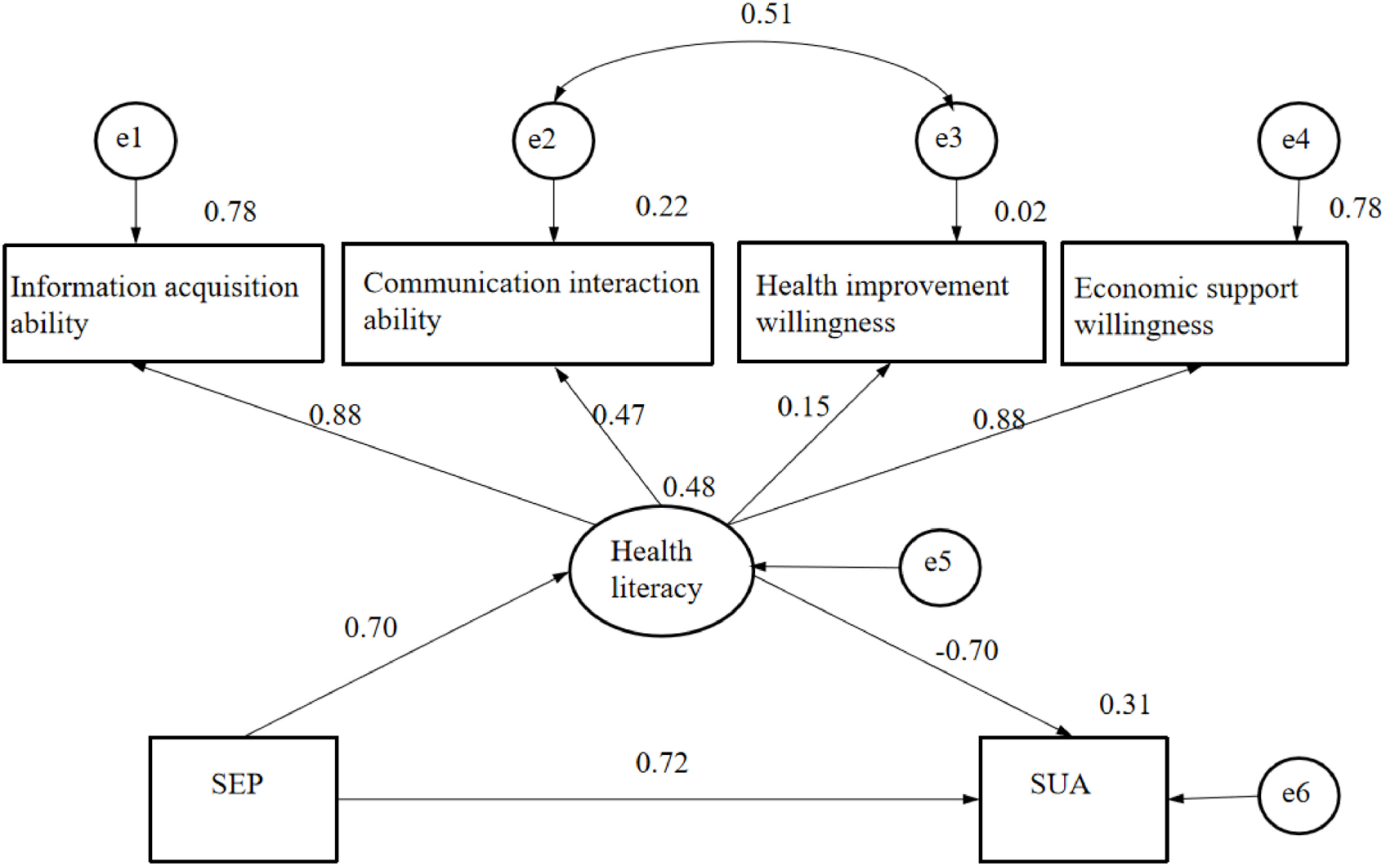
Final model and standardized pathway coefficients for health literacy, SEP, and SUA levels

The model revealed the association between SEP and SUA levels and the effect of health literacy on this association. Figure 3 and Table 6 show all of the estimated direct effects from path analyses. The results of significance testing of the mediating pathways showed that health literacy mediated the influence of SEP on SUA levels. In AHU patients, a one-unit increase in socioeconomic status was associated with a 2.561-unit increase in health literacy and an increase of 41.329 μmol/L in the SUA level. However, an increase of one unit in the health literacy of the patients was linked to a decrease of 11.133 μmol/L in the SUA level. SEP had a direct positive effect on SUA levels (*β* = 0.723) and had an indirect effect through health literacy (*β* = -0.490). The total effect of SEP on SUA levels was 0.233 (95% CI: 0.130 to 0.336).

**Table 6:** Path analysis results of SUA levels in patients with AHU

Model pathways	Estimate	Standardized Estimate	S.E	95% CI
Total effect SEP → SUA	13.315*	0.233	2.885	(0.130,0.336)
Direct effect SEP → SUA	41.329*	0.723	4.004	(0.606, 0.859)
Indirect effect SEP → health literacy → SUA	-28.014*	-0.490	3.645	(-0.620, -0.382)
SEP → health literacy	2.561*	0.696	0.169	-
Health literacy → SUA	-11.133*	-0.704	1.220	-

*SEP* Socioeconomic position, *SUA* Serum uric acid, *S.E.* Standard error in estimate; * P < 0.001

## Discussion

This study is the first to investigate the health literacy status of patients with AHU in China, and the results showed that 155 (44.4%) of the subjects had basic health literacy. In addition, this is the first study in which, to our knowledge, an SEM was constructed to examine the associations between health literacy, SEP, and SUA levels. The findings showed that health literacy plays a mediating role in the relationship between SEP and SUA levels.

In this study, the mean health literacy score of the AHU patients was 90.18 $\pm$ 15.11, and 55.6% of the patients had deficient health literacy. Because there is no health literacy scale for AHU patients and health literacy has not yet been investigated in AHU patients, this study used the tool for patients with chronic diseases, which made it difficult to make a horizontal comparison. Studies have shown that chronic metabolic diseases such as HUA, diabetes, and hypertension have many common causes [39, 40]. Diabetes and hypertension are the main chronic diseases in China, and HUA management needs to learn from them. These results showed that the AHU patients had lower health literacy survey scores than hypertension patients (91.57 ± 7.38) [41] but higher scores than diabetes patients (88.83 ± 12.57) [42]. Considering that the patients volunteered to participate, we offered incentives, which may have caused an overestimation of health literacy. Therefore, the majority of AHU patients lack health literacy, which might be due to the absence of clinical manifestations of AHU and lack of awareness and management of related diseases, even among primary health care physicians [43]. The greater an individual’s health literacy is, the better their self-management ability. Thus, it is important to further improve the health literacy level of AHU patients, strengthen continuing education, and reinforce the management of chronic diseases caused by AHU for primary healthcare physicians.

The results of our study revealed that health literacy was negatively related to SUA levels. This is likely because patients with adequate health literacy and greater health awareness are more likely to proactively search for health information. Patients might have a greater acceptance of knowledge and be more likely to communicate effectively with medical staff, so they better manage their SUA levels. A study revealed that enhancing health literacy is a possible intervention target for improving patients' self-care behaviours [43]. Individuals with higher health literacy are more likely to perform health behaviours by using health information, paying attention to health status, and having more confidence in managing their SUA levels. Other factors may also contribute to the differences in SUA levels. For example, a healthy lifestyle is beneficial for decreasing SUA levels. Even though other factors may play a role, our study still indicated that health literacy may serve as a key predictor of self-management of SUA levels among patients with AHU. Health literacy includes four core elements: knowledge, attitude, skill, and behaviour. Education must be transformed into cognition and attitude and then implemented through skills and behaviours. Health literacy is the result of understanding and utilizing health-related information. In the case of patients with AHU, health literacy presents the ability and channels to capture knowledge to some extent, which in turn act on patients’ behaviours, efforts, and management of SUA levels.

In addition, we found that high SEP was associated with high SUA levels, which is consistent with the findings of a study in the Korean population [44]. This may be related to individuals’ dietary habits and lifestyles. Patients with higher SEP have easier access to high-protein foods (i.e., beef, pork, and lamb) due to better economic conditions, which can lead to the intake of excess purine [27]. Moreover, individuals with a high SEP usually consume more alcohol during interpersonal communication, which might be related to Chinese food culture. Previous research has shown that consuming meat or seafood and consuming alcohol increases the risk of HUA [45, 46]. Patients with higher education and occupational prestige are mostly sedentary workers and are less physically active [47]. Research shows that the likelihood of AHU is greater in adults who spend ≥ 10 h per day than in those who spend < 5 h per day engaging in sedentary behaviour [48]. In addition, AHU patients with high SEP are more concerned about their health and have the ability to obtain more health resources, increasing the ease of identification during routine physical examinations.

The mean score for social support was 42.15 $\pm$ 7.00, which showed that more than half of AHU patients received medium social support (56.4%). The results were lower than AHU patients surveyed by Liu XF (44.84 $\pm$ 6.73) [26]. The reason for this difference might be that the patients in our study majority were middle-aged and elderly people (63.3%), while the study population of Liu XF was young and middle-aged. Abu-Kaf et.al found that older people's social support was mainly from family members, and they acquired support from friends, organizations, and communities less [49]. This is consistent with Zhou J's study of elderly people in China [50]. Prior research findings showed that increased social support was related to lower SUA levels [51]. Thus, sufficient social support is needed for AHU patients, especially elderly patients.

In our study, the zero-order correlations showed that social support was significantly positively correlated with SUA levels, but the correlation was not significant after controlling for age, gender, and AHU duration. This finding suggested that confounding factors contribute more to SUA levels than does social support. For example, patients who have longer durations of AHU might have a better understanding of the disease and better living habits. Through long-term treatment and disease management, SUA levels might eventually change. Research by Thomas et al. indicated that individuals with high social support levels had lower SUA levels [22]. The reason might be that the participants in our study were AHU patients, while Thomas’s study focused on elderly individuals. In addition, we surmise that the lack of an explanatory relationship between social support and SUA levels could be related to the social support measurement used in this study. The social support score is based on patients’ self-reports and is influenced by their subjective consciousness. The association between social support and SUA levels is complex and might be affected by many confounding factors, such as patient self-efficacy. Thus, this study did not include social support as a mediating variable in the SEM. However, the effectiveness of social support in improving health outcomes is increasingly obvious [52]. Additionally, the finding that higher social support levels were linked to improved health literacy is consistent with the findings of a prior study [53]. Social support has a mediating role in the relationship between health literacy and self-rated health [54]. Research indicates that individuals with low health literacy often conceal their issues from loved ones and friends due to feelings of shame or guilt [55]. Those with poor health literacy might tend to isolate themselves, lack social interaction, and fear communicating with medical staff. However, individuals with high health literacy may access health information through various channels and be willing to share their knowledge with others, which promotes better mental and physical health. Given the modifiable nature of social support, different interventions can be taken to enhance social support, such as education, physical exercise, and group activities. Future research should focus on understanding how interventions might be applied to reduce health disparities.

An important finding of this study was that the relationship between SEP and SUA levels was partially mediated by health literacy, and the indirect mediating effect of SEP on health literacy was -0.490, signifying that health literacy had a negative mediating effect on the relationship between SEP and SUA levels in this model. Research has shown that SEP could improve health status by increasing health literacy [56, 57]. These results were consistent with the findings of the present study, which showed that the higher an individual’s SEP was, the greater their health literacy, and the lower their SUA levels. The conceptual model of health literacy proposed by Kristine [58] can explain the relationships between health literacy, SEP, and SUA levels. Three primary modules comprise this framework: (1) antecedents of health literacy (e.g., societal and environmental determinants, personal determinants, and situational determinants); (2) the main dimensions of health literacy, which consists of health care, disease prevention and health promotion domains; and (3) consequences of health literacy (e.g., health behaviours, health outcomes). Socioeconomic factors such as education level, income, and occupation type can affect individuals’ cognitive, behavioural, and social skills, which are the proximal factors affecting health literacy [59]. Health literacy is a phenomenon that has positive consequences and, in turn, influences patients’ health behaviours, thereby changing lifestyles and controlling SUA levels. Health literacy and social support are more easily modified than SEP, and we can implement health education and health promotion strategies to help patients understand health-related information, thereby enhancing their health literacy and improving their health status [60]. First, family members should provide more social support (both financial and emotional support) for AHU patients and provide them with more knowledge about health care. Second, primary healthcare providers should evaluate health literacy status during initial meetings with AHU patients and emphasize its significance during follow-up visits. Third, community and health care centers could provide extensive health lectures and health knowledge activities to help optimize patients’ understanding and improve their health literacy and social support.

Our study has several limitations. First, this was a cross-sectional study, and the causal relationships between SEP and social support, health literacy, and SUA levels were not validated. Second, the sample in this study was selected mainly from the community using a convenience sampling method. Moreover, the sample included mostly middle-aged and elderly people, which limits generalizability. Third, SEP indicators (education level, income, and occupation type) were self-reported by the participants, which may be influenced by their subjective perceptions and may have led to the overestimation or underestimation of the results. Fourth, the HeLMS used in this study measured general chronic health literacy rather than HUA-specific health literacy. Future research could use a larger random sample and interventions to determine the associations between SEP and health literacy and between social support and SUA levels as well as to explore the multiple mediating roles of variables, such as lifestyle factors, on health outcomes among patients with AHU. In addition, developing a health literacy scale for AHU patients is necessary.

## Conclusion

In conclusion, our study indicated that AHU patients with low health literacy, and health literacy might constitute one of the potential pathways through which socioeconomic factors impact SUA levels. However, social support was not significantly associated with SUA levels. Measures should be taken to improve health literacy and social support to enhance patient self-management and health status. In addition, implementing health literacy screening and tailored interventions for patients will ultimately contribute to improving health literacy and decreasing SUA levels, especially for patients with a lower SEP.

### Supplementary Information

Supplementary Material 1.

## Data Availability

The datasets used and/or analysed during the current study are available from the corresponding author upon reasonable request.

## Abbreviations

AHU: Asymptomatic hyperuricaemia
HUA: Hyperuricaemia
HLSCP: Health Literacy Scale for Chronic Patients
KMO: Kaiser–Meyer–Olkin
PCA: Principal component analysis
SEM: Structural equation modelling
SEP: Socioeconomic position
SSRS: Social Support Rating Scale
SUA: Serum uric acid
