# Supplementary material for: Impact of health literacy, social support, and socioeconomic position on the serum uric acid level in asymptomatic hyperuricaemia patients in China: a structural equation model
Source: BMC Public Health. 2024 Jun 17;24:1606. doi: 10.1186/s12889-024-19085-6 (PMC11181562; doi:10.1186/s12889-024-19085-6)
Supplement: Supplementary file 1 — Supplementary Material 1. [file 12889_2024_19085_MOESM1_ESM.pdf]

Additional file 1

Questionnaire:

Health Literacy Survey for Hyperuricemia Patients

Personal Basic Information

1. Gender: ① Male ② Female

2. Age: _____years old

3. Height: _____cm

4. Weight: _____ kg

5. Family Residence: ① Rural ② Urban

6. Residency Pattern: ① Solo residence ② Not solo residence

7. Marital Status: ① Married ② Single ③ Divorced ④ Widowed

8. Education Level: ① Primary School or Below ② Junior High School ③ High School or Technical School ④ College ⑤ Bachelor's Degree or above

9. Occupation: ① Government official ② Teacher ③ Medical staff ④ Other staff in the public institution ⑤ Student ⑥ Farmer ⑦ Worker ⑧ Other enterprise staff ⑨ Other_____

10. Monthly income per household member：

① Less than or equal to 2,000 RMB ② 2,001 to 3,000 RMB ③ 3,001 to 4,000 RMB ④ 4,001 to 5,000 RMB ⑤ More than 5,000 RMB

11. The ability to pay for family medical costs:

① Unable to pay ② Can barely pay ③ Able to pay

Conditions associated with hyperuricemia

12. Do you have the following comorbidities (you can have multiple choices):

① Diabetes ② Hypertension ③ Cardiovascular and cerebrovascular diseases

④ Kidney disease ⑤ Fatty liver ⑥ Hyperlipidemia ⑦ Others__________

13. Duration of hyperuricemia: _____years

14. Last blood uric acid detection level: ______ μ mol/L

15. Whether taking uric acid-lowering drugs: ①Yes ②No

Social Support Rating Scale

1. How many close friends do you have, from whom you can receive support and help?

① devil a one ② one or two

③ three to five ④ six or more

2. Recent one year：

① stay away from family and live alone.

② moved around a lot, lived with strangers most of the time.

③ live with classmates, colleagues or friends.

④ live with family members.

3. You and neighbors:

① never care about each other, just nodding acquaintance.

② may be a little concerned when it comes to difficulties.

③ some of the neighbors are concerned about you.

④ most of the neighbors are concerned about you.

4. You and colleagues：

① never care about each other, just nodding acquaintance.

② may be a little concerned when it comes to difficulties.

③ some of the colleagues are concerned about you.

④ most of the colleagues are concerned about you.

5. From whom can you receive support and help? (Tick " √ " in the appropriate box)

	no	seldom	General	Full support
A. Couple				
B. Parents				
C. Children				
D. Brothers and sisters				
E. Others				

6. In the past, when you encountered difficulties, what was your source of financial support and help to solve practical problems?

① No source

② Following sources (you can have multiple choices):

A. Spouse B. Other family members C. Friends D. Relatives E. Colleagues F. Work unit G. Official or semi-official organizations H. Religion, social organization, and other non-official organizations I. Others_________

7. In the past, when you were in an emergency situation, the sources of comfort and concern that you received were :

① No source

② Following sources (you can have multiple choices):

A. Spouse B. Other family members C. Friends D. Relatives E. Colleagues F. Work unit G. Official or semi-official organizations H. Religion, social organization, and other non-official organizations I. Others_________

8. Who do you ask for help when you encounter trouble?

① Never told anyone ② Only speak to 1-2 people who are very close to you

③ If a friend asks, you'll tell them ④ Take the initiative to talk about troubles for support and understanding

9. What kind of help do you choose when you are in trouble?

① I rely solely on myself and refuse to accept any help from others.

② I rarely ask for help from other .

③ I sometimes ask for help from others.

④ I often seek assistance from family, friends, organizations, and other groups when I encounter difficulties.

10. Regarding group activities organized by organizations such as party organization, religious groups, trade unions, student associations, etc., you:

① Never participate ② Occasionally participate

③ Often participate ④ Volunteer and actively engage in activities

Health Literacy Scale for Chronic Patients[1]

Item	Completely cannot	Very difficult	Some difficulty	Few difficulty	No difficulty
1） Read health information brochures	1	2	3	4	5
2）Make health decisions	1	2	3	4	5
3）Read written health information	1	2	3	4	5
4）Understand health information	1	2	3	4	5
5）Follow doctor’s instructions	1	2	3	4	5
6）See doctors independently	1	2	3	4	5
7）Communicate with doctors	1	2	3	4	5
8）Pay for medical treatment	1	2	3	4	5
9）Fill in medical blanks independently	1	2	3	4	5
10）Pay for managing your own health	1	2	3	4	5
11）Find needed health information	1	2	3	4	5
**Item**	**Never**	**Seldom**	**Sometimes**	**Usually**	**Always**
12) Seek the unknown health problem actively	1	2	3	4	5
13）Ask family or friends for help	1	2	3	4	5
14）Join in some activities with other patients	1	2	3	4	5
15）Prepare before seeing the doctor	1	2	3	4	5
16）Discuss health with others	1	2	3	4	5
**Item**	**Have never thought**	**Thought but did not act**	**Seldom**	**Occasionally**	**Often**
17）Put health information into	1	2	3	4	5

practice					
18）Ask doctors something unknown	1	2	3	4	5
19）Follow up doctors to understand something	1	2	3	4	5
20）Ask family or friends accompany you to see a doctor	1	2	3	4	5
**Item**	**Very reluctant**	**Reluctant**	**Not matter**	**Willing**	**Very willing**
21）Willing to make time for health	1	2	3	4	5
22）Willing to pay attention to your health need	1	2	3	4	5
23）Willing to find the energy to manage health	1	2	3	4	5
24）Willing to change unhealthy life style	1	2	3	4	5

investigator：_____

Reference

[1] Sun H, Peng H, Fu H: The reliability and consistency of health literacy scale for chronic patients. Fudan University Journal of Medical Sciences. 2012;39(03):268-272.
